# Increased estimated remnant-like particle cholesterol is associated with impaired coronary collateralization in patients with coronary chronic total occlusions

**DOI:** 10.1186/s13098-022-00829-6

**Published:** 2022-04-21

**Authors:** Ang Gao, Jinxing Liu, Yan Liu, Chengping Hu, Yong Zhu, Yujie Zhou, Hongya Han, Yingxin Zhao

**Affiliations:** 1 Department of Cardiology, Beijing AnZhen Hospital, Capital Medical University, Beijing, 100029 China; 2 Department of Cardiology, National Center for Cardiovascular Diseases, Fuwai Hospital, Chinese Academy of Medical Sciences and Peking Union Medical College, Beijing, China

**Keywords:** Remnant-like particle cholesterol, Coronary chronic total occlusion, Coronary collateralization, Coronary artery disease, Type 2 diabetes mellitus

## Abstract

**Aims:**

This study intends to explore whether, or to what extent, the estimated remnant-like particle cholesterol was associated with coronary collateralization in patients with chronic total occlusion lesions.

**Methods:**

792 patients with at least one coronary chronic total occlusion lesion were enrolled. Serum level of lipid profiles were determined and the estimated remnant-like particle cholesterol was calculated. The development of coronary collateralization was graded as low (Rentrop score 0–1) or high (Rentrop score 2–3) collateralization according to the Rentrop classification system and then the association between the estimated remnant-like particle cholesterol and collateralization was assessed.

**Results:**

222 participants were classified into low collateralization group. The estimated remnant-like particle cholesterol level was significantly higher in low collateralization (*P* < 0.001) and type 2 diabetes mellitus (*P* = 0.009) group. To further explore the association between the estimated remnant-like particle cholesterol and the development of coronary collateralization, these patients were divided into 3 groups based on the estimated remnant-like particle cholesterol tertiles. The prevalence of low collateralization increased stepwise with the tertile groups (T1 12.5% *vs.* 27.1% *vs.* 45.3%, *P* < 0.001). Multivariate logistic regression analysis showed that the estimated remnant-like particle cholesterol was independently associated with the under-developed collateralization, with an OR and 95%CI of 2.34 (1.46–3.74) and 4.91 (3.01–8.02) in the T2 and T3 group, respectively. The following receiver-operating characteristic analysis indicated that the diagnostic value of estimated remnant-like particle cholesterol for the low collateralization was 0.696, with a cut-off value of 0.485, and its sensitivity was 82.88%. Besides, the addition of the estimated remnant-like particle cholesterol into the baseline model consisting of traditional risk factors could improve the incremental value of the discrimination of impaired collateralization only in overall and type 2 diabetes mellitus populations.

**Conclusions:**

The increased estimated remnant-like particle cholesterol is independently associated with impaired collateralization in patients with coronary chronic total occlusion lesions. Therapies targeting at remnant-like particle cholesterol may be needed in advanced coronary artery disease patients with type 2 diabetes mellitus not suitable for vascular revascularization.

## Introduction

Coronary chronic total occlusion (CTO) lesions refer to the coronary lesions that are completely occluded with Thrombolysis In Myocardial Infarction (TIMI) 0 flow with an estimated duration of at least 3 months and are serious clinical presentations of coronary artery disease (CAD) and need to take due attention [1]. The success rate of recanalization of CTO lesions increased steadily owing to growing operator experience, technological improvement and evolving techniques [2]. However, to date whether invasive strategy of CTO lesions could improve the prognosis of those patients remained to be controversial. Recently, a meta-analysis compared the treatment efficacy of percutaneous coronary intervention (PCI) and optimal medical therapy alone and found that PCI could not improve long-term survival or decrease major adverse cardiac events (MACEs) rates in those patients [3]. Besides, CTO-related PCI is also associated with a higher periprocedural risk. A recent trial focusing on chronic kidney disease (CKD) patients with CTO lesions undergoing PCI showed that those patients suffered higher all-cause death rates whereas the technical and procedural success rates are significantly lower than those without CKD [4]. Hence, invasive revascularization strategy may not be the optimal option for the management of those advanced CAD patients with certain complications. Coronary collateral vessels are interarterial connections between major epicardial arteries and could compensate for myocardial ischemia and improve clinical outcomes in the event of coronary artery obstruction [5]. The mechanism underlying coronary collateral growth (CCG) is complex. When one major epicardial artery is severely obstructed, increased fluid shear stress caused by elevated pressure gradient along the arterial network would activate endothelial cells and monocytes secreting proangiogenic factors to promote collateral growth [6]. However, it remains elusive why some CAD patients, especially those with Type 2 diabetes mellitus (T2DM) or metabolic syndrome (MS), tend to form less developed collateralization [7, 8].

Remnant-like particle cholesterol (RLP-C), defined as the cholesterol content of triglyceride (TG) rich lipoproteins (TRL) consisting of very low-density lipoprotein and intermediate-density lipoprotein particles, has been recognized as a residual atherosclerotic cardiovascular disease (ASCVD) risk [9]. Increasing clinical evidence supported that RLP-C is involved in the progression of atherosclerosis and some even pointed out that it’s more atherogenic than low-density lipoprotein cholesterol (LDL-C) [10]. Former population studies have found RLP-C is associated with low-grade inflammation, which is thought to be the biochemical mechanism underlying the formation of impaired collateralization [11–13]. T2DM is characterized by impaired lipid metabolism and the RLP-C has been substantiated to be more prominent in those T2DM patients [14]. Considering the importance of early detecting less developed collateralization in CAD patients with CTO lesions and certain clinical implication (such as T2DM and MS) as MACEs rates are significantly higher in those patients partly due to impaired collateralizations [15, 16]. Hence, it’s pertinent to explore the association of the RLP-C and coronary collaterals in CAD patients especially for metabolic abnormality patients with CTO lesions.

## Methods

### Study population

Patients undergoing cardiac catheterization and later substantiated as coronary CTO lesions at Beijing AnZhen Hospital between 1st January 2020 and 31st December 2020 were recruited into this single-center observational study. The estimation of the duration of the occlusion was based on the occurrence of myocardial infarction (MI) related to the occluded vessel or the comparison with prior angiogram. For the purpose of this study, the exclusion criteria were as followed: (1) history of coronary artery bridge grafting; (2) severe renal and hepatic dysfunction; (3) New York Heart Association (NYHA) III-IV or left ventricular ejection fraction (LVEF) < 30%; (4) Type 1 Diabetes Mellitus; (5) suspected familial hypertriglyceridemia; (6) Infectious or malignant disease; (7) some basic demographic data and lipid profiles were not available; Overall, 792 participants were enrolled in this study.

### Disease definition

The diagnosis of hypertension (HTN) is based on the recommendations from the European Society of Cardiology/European Society of Hypertension [17]: Office systolic blood pressure ≥ 140 mmHg and/or diastolic blood pressure ≥ 90 mmHg or the history of antihypertensive agents in the past 2 weeks. The diagnosis of T2DM comes from the previous diagnosis and recommendations from American Diabetes Association [18]: 1) Fasting blood glucose (FBG) ≥ 126 mg/dL (7 mmol/L); 2) 2 h-postprandial glucose ≥ 200 mg/dL (11.0 mmol/L) during 75 g oral glucose tolerance test; 3) Glycated hemoglobin A1c (HbA1c) ≥ 6.5%; 4) Classic symptom of hyperglycemia or hyperglycemic crisis, a random plasma glucose ≥ 200 mg/dL (11.1 mmol/L). There has always been considerable disparity over the components and criteria of the MS, especially in different ethnic groups. Several diagnostic criteria for MS are now applied in clinical practice, such as International Diabetes Federation (IDF), the National Cholesterol Education Program Third Adult Treatment Panel (NCEP ATP III) and American Heart Association/National Heart, Lung, and Blood Institute (AHA/NHLBI) criterion, and so on[19]. The current study mainly enrolled Chinses populations with cardiovascular diseases (CVD), so a criterion of MS would be better if it’s more suitable for the evaluation of CVD risks in a large Chinese population. A recent study conducted by Yu et al. [20] enrolled 2486 patients from rural China to evaluate the predictive value of MS for newly onset CVD and showed that the NECP ATP III criteria to define MS is more accurate to predict the incidence of CVD. Hence, MS is defined according to the guideline for NCEP ATP III. The current study used body mass index (BMI) as an alternative for waist circumference (WC) because of the lack of data about WC, including 3 or more following components can be seen as abnormalities[21]: 1) systolic blood pressure ≥ 130 mmHg, diastolic blood pressure ≥ 85 mmHg; 2) high TG > 150 mg/dL; 3) low HDL-C (male < 40 mg/dL, female < 50 mg/dL); 4) elevated fasting glucose ≥ 110 mg/dL (6.1 mmol/L) or a history of T2DM; 5) overweight or obesity (BMI ≥ 25 kg/m^2^).

### Demographic and laboratory measurements

Demographic data was collected from Beijing AnZhen hospital medical information record system. LVEF is calculated by echocardiography at admission. Blood samples were taken after an overnight fasting and were sent to accepting standard technical measurements. The non-HDL-C was calculated as TC minus HDL-C and the estimated RLP-C was determined by the formula subtracting LDL-C and HDL-C from TCHO.

### Coronary angiography and rentrop classification system

The development of coronary collateralization was evaluated by cardiac catheterization and the results of angiogram were assessed by two experienced interventional cardiologists who were blinded for this study. Rentrop scoring system was used to assess the grading of coronary collaterals: Grade 0: no visible filling of any collateral vessel; Grade 1: filling of the side branch via collateral channel but without filling of the epicardial arteries; Grade 2: partial filling of the epicardial artery via collateral branches; Grade 3: complete filling of the epicardial artery [22]. In patients with multi-collateral branch, the highest grading was selected into the final analysis. Coronary collateral (Rentrop score 0–1) was defined as low collateralization, also can be termed as impaired or less developed collateralization.

### Statistical analysis

Of the 792 participants enrolled in this study, we further classified them into low and high collateralization group according to the collateral gradings. Data were expressed as mean ± standard deviation, except for FBG, glycated albumin (GA), high-sensitivity C-reactive protein (hs-CRP) and lipoprotein (a). The distributions of markers are highly skewed; Those indicators were expressed as the median and interquartile range and transformation into natural logarithms before statistical analysis. The Difference between the two groups were compared by Students’ t test or Mann-Whiteney U test for normally or non-normally distributed variables. Multivariate logistic regression analysis was used to evaluate the association between the estimated RLP-C and the formation of collateralizations. Finally, to evaluate the diagnostic value of the estimated RLP-C for impaired collateralization, the area under the curve (AUC) and optimal cut-off value was assessed through receiver-operating characteristic (ROC) analysis. De Long test was used to compared the AUCs between different models. We further compare the risk efficacy value of the estimated RLP-C in populations with different glycometabolic status. Integrated discrimination index (IDI) and net reclassification index (NRI) were calculated to evaluate the incremental risk assessment effect of the estimated RLP-C in patients with T2DM or not. All statistical analysis mentioned above were conducted using SPSS 23.0 (SPSS, Inc., Chicago, IL, USA), R project (version 4.0.5) and MedCalc Statistical Software (version 19.1 Ostend, Belgium). Two-sided *P* value < 0.05 was considered statistically significant.

## Results

### Baseline characteristics

The demographic, laboratory and angiographic characteristics of participants were shown in Table 1. The mean age of those patients 58.97 ± 10.27 years old and most of them were males (85.5%). Most patients come with one or more cardiovascular risk factors, the prevalence of current smokers, HTN, T2DM, and MS were 55.3% ,68.8%, 42.8%, and 60.4%, respectively. Moreover, no difference in age, gender, BMI, smoking status and hypertension were found between low and high collateralization groups. Compared with high collateralization groups, the impaired ones had more rate of MS (*P* < 0.001). Though not statistically significant, the prevalence of T2DM rates seemed to be higher in the low collateralization group (*P* = 0.066). As for laboratory measurements, patients with high collateralization have lower lipid profiles, glycometabolic and inflammatory markers (*P* < 0.05). As with angiographic characteristics, most of patients are diagnosed as multivessel disease (86%). Coronary angiographic analysis indicated that right coronary artery (RCA) -related CTO lesions are prone to form good collaterals while left anterior descending artery (LAD) -related CTO lesions tend to develop impaired coronary collateral branches.

**Table 1:** Baseline clinical characteristics of patients stratified by collateral grading

	Total (n = 792)	Low collateralization	High collateralization	*P* values
		(n = 222)	(n = 570)	
Demographic data				
Age (years)	58.97 ± 10.27	58.51 ± 9.93	59.15 ± 10.40	0.437
Male sex, n (%)	677 (85.5%)	184 (82.9%)	493 (86.5%)	0.217
BMI (Kg/m^2^)	26.39 ± 3.27	26.62 ± 3.09	26.30 ± 3.33	0.229
SBP (mmHg)	130.55 ± 16.59	130.22 ± 16.75	130.68 ± 16.53	0.724
DBP (mmHg)	76.95 ± 11.11	77.10 ± 10.93	76.89 ± 11.19	0.805
Current Smokers, n (%)	438 (55.3%)	128 (57.7%)	310 (54.4%)	0.427
Medical history				
Hypertension, n (%)	545 (68.8%)	148 (66.7%)	397 (69.6%)	0.442
T2DM, n (%)	339 (42.8%)	107 (48.2%)	232 (40.7%)	0.066
MS, n (%)	478 (60.4%)	165 (74.3%)	313 (54.9%)	< 0.001*
Laboratory measurement				
eGFR	90.93 ± 16.97	90.06 ± 18.57	91.27 ± 16.31	0.395
CREA, mmol/L	79.01 ± 20.26	80.16 ± 22.64	78.56 ± 19.25	0.353
UREA, mmol/L	5.60 ± 1.90	5.79 ± 2.03	5.53 ± 1.84	0.082
UA, mmol/L	360.23 ± 96.10	368.56 ± 96.60	356.98 ± 95.79	0.128
FBG, mmol/L	5.83 (5.08–7.29)	6.41 (5.24–8.21)	5.69 (5.04–6.96)	< 0.001*
GA, %	14.41 (12.96–17.24)	14.54 (13.00–18.23)	14.39 (12.93–16.83)	0.142
HbA1C, %	6.52 ± 1.22	6.76 ± 1.33	6.43 ± 1.16	0.001*
TCHO, mmol/L	3.90 ± 1.01	4.14 ± 1.08	3.80 ± 0.96	< 0.001*
TG, mmol/L	1.76 ± 0.89	2.26 ± 1.07	1.56 ± 0.72	< 0.001*
HDL-C, mmol/L	1.03 ± 0.24	0.98 ± 0.23	1.05 ± 0.24	0.001*
LDL-C, mmol/L	2.25 ± 0.89	2.37 ± 0.94	2.20 ± 0.86	0.016*
Non-HDL-C, mmol/L	2.86 ± 0.98	3.15 ± 1.06	2.75 ± 0.92	< 0.001*
Lp (a), g/L	0.14 (0.06–0.41)	0.15 (0.06–0.49)	0.14 (0.06–0.38)	0.027*
Estimated RLP-C, mmol/L	0.61 ± 0.32	0.78 ± 0.39	0.55 ± 0.27	< 0.001*
HCY, μmol/L	13.80 (11.80–18.15)	13.90 (11.88–19.55)	13.80 (11.78–17.90)	0.370
Hs-CRP, mg/l	1.02 (0.53–2.51)	1.13 (0.66–3.21)	0.99 (0.51–2.33)	0.030*
FFA, mmol/L	0.45 ± 0.22	0.46 ± 0.20	0.45 ± 0.22	0.661
LVEF, (%)	59.03 ± 8.98	59.35 ± 9.06	58.90 ± 8.96	0.555
Cardiovascular Medication				
Antiplatelet therapy, n (%)	685 (86.5%)	191 (86.0%)	494 (86.7%)	0.818
β-blockers, n (%)	489 (61.7%)	142 (64.0%)	347 (60.9%)	0.464
ACEI/ARB, n (%)	270 (34.1%)	85 (38.3%)	185 (32.5%)	0.133
Statins, n (%)	660 (83.3%)	187 (84.2%)	473 (83.0%)	0.750
CCB, n (%)	232 (29.3%)	59 (26.6%)	173 (30.4%)	0.339
Coronary angiography data				
Severity of CAD				0.758
One-vessel disease, n (%)	111 (14.0%)	27 (12.2%)	84 (14.7%)	0.425
Two-vessel disease, n (%)	220 (27.8%)	60 (27.0%)	160 (28.1%)	0.792
LM/Three-vessel disease, n (%)	461 (58.2%)	135 (60.8%)	326 (57.2%)	0.378
CTO related artery				0.575
RCA, n (%)	376 (47.5%)	100 (45.0%)	276 (48.4%)	0.428
LCX, n (%)	149 (18.8%)	41 (18.5%)	108 (18.9%)	0.920
LAD, n (%)	267 (33.7%)	81 (36.5%)	186 (32.6%)	0.316
ISR-CTO, n (%)	74 (9.3%)	28 (12.6%)	46 (8.1%)	0.057

^*^indicated the difference between groups is statistically significant

*BMI* Body mass index, *SBP* Systolic blood pressure, *DBP* Diastolic blood pressure, *T2DM* Type 2 diabetes mellitus, *MS* Metabolic syndrome, *eGFR* Estimated glomerular filtration rate, *CREA* Creatinine, *UREA* Urea, *UA* Uric acid, *FBG* Fasting blood glucose, *GA* Glycated albumin, *HbA1c* Glycosylated hemoglobin A1c, *TC* Total cholesterol, *TG* Triglyceride, *HDL-C* High-density lipoprotein cholesterol, *LDL-C* Low-density lipoprotein cholesterol, *non HDL-C* Non high-density lipoprotein cholesterol, *Lp(a)* Lipoprotein(a), *sd LDL-C* Small dense low-density lipoprotein cholesterol, *RLP-C* Remnant-like particle cholesterol, *hs-CRP* High sensitivity C-reactive protein, *FFA* Free fatty acid, *LVEF* Left ventricular ejection fraction, *ACEI* Angiotensin-converting enzyme inhibitor, *ARB* Angiotensin receptor blocker, *CCB* Calcium channel blocker, *CAD* Coronary artery disease, *CTO* Chronic total lesion, *RCA* Right coronary artery, *LCX* Left circumflex artery, *LAD* Left anterior descending artery, *ISR* In-stent restenosis

### Association between RLP-C and coronary collateralization

**Fig. 1:**
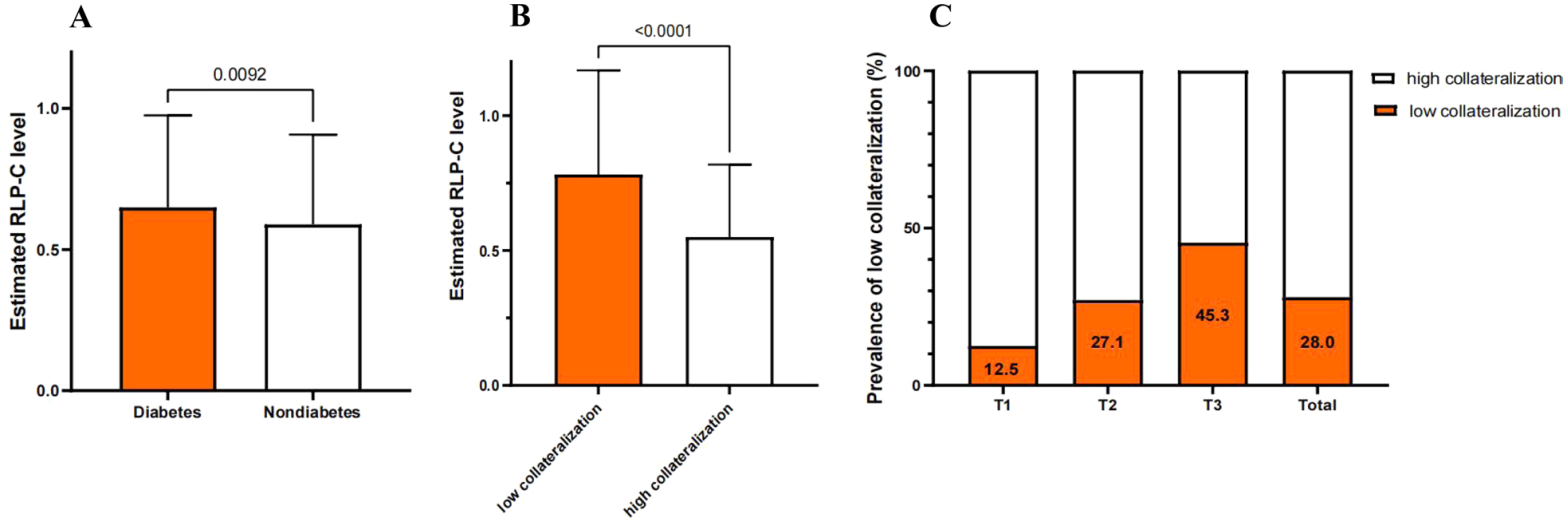
Comparison of the estimated RLP-C level between T2DM groups or not;** A** Comparison of the estimated RLP-C level between the low collateralization and high collateralization group;** B** prevalence of low collateralization according to the RLP-C tertiles. ** C** *RLP-C* Remnant-like particle cholesterol

Figure 1A and B showed the estimated RLP-C level is significantly higher in low collateralization (*P* < 0.001) and T2DM patients (*P* = 0.009). Figure 1C indicated that the proportion of low collateralization increased stepwise from the lowest one to the highest once dividing these patients into 3 tertiles according to the estimated RLP-C level (*P* < 0.001).

**Fig. 2:**
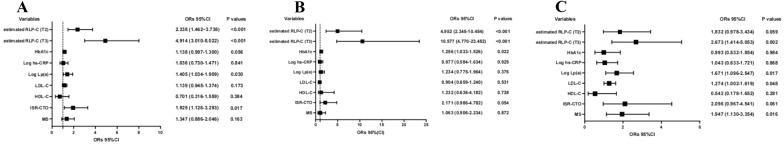
Forest plot of the multivariate logistic regression analysis model in patients with CTO lesions exploring the association between various risk factors and low collateralization. Overall population (**A**); T2DM ( +) group (**B**); T2DM ( − ) group (**C**). *CTO* Chronic total occlusion, *RLP-C* Remnant-like particle cholesterol, *T2DM* Type 2 diabetes mellitus, *MS* Metabolic syndrome, *Lp(a)* Lipoprotein(a), *Hs-CRP* High-sensitivity C-reactive protein, *LDL-C* Low-density lipoprotein cholesterol, *HDL-C* High-density lipoprotein cholesterol, *HbA1c* Glycated hemoglobin A1c, *ISR* In-stent restenosis, *ORs* Odds ratios, *CI* Confidential interval

**Fig. 3:**
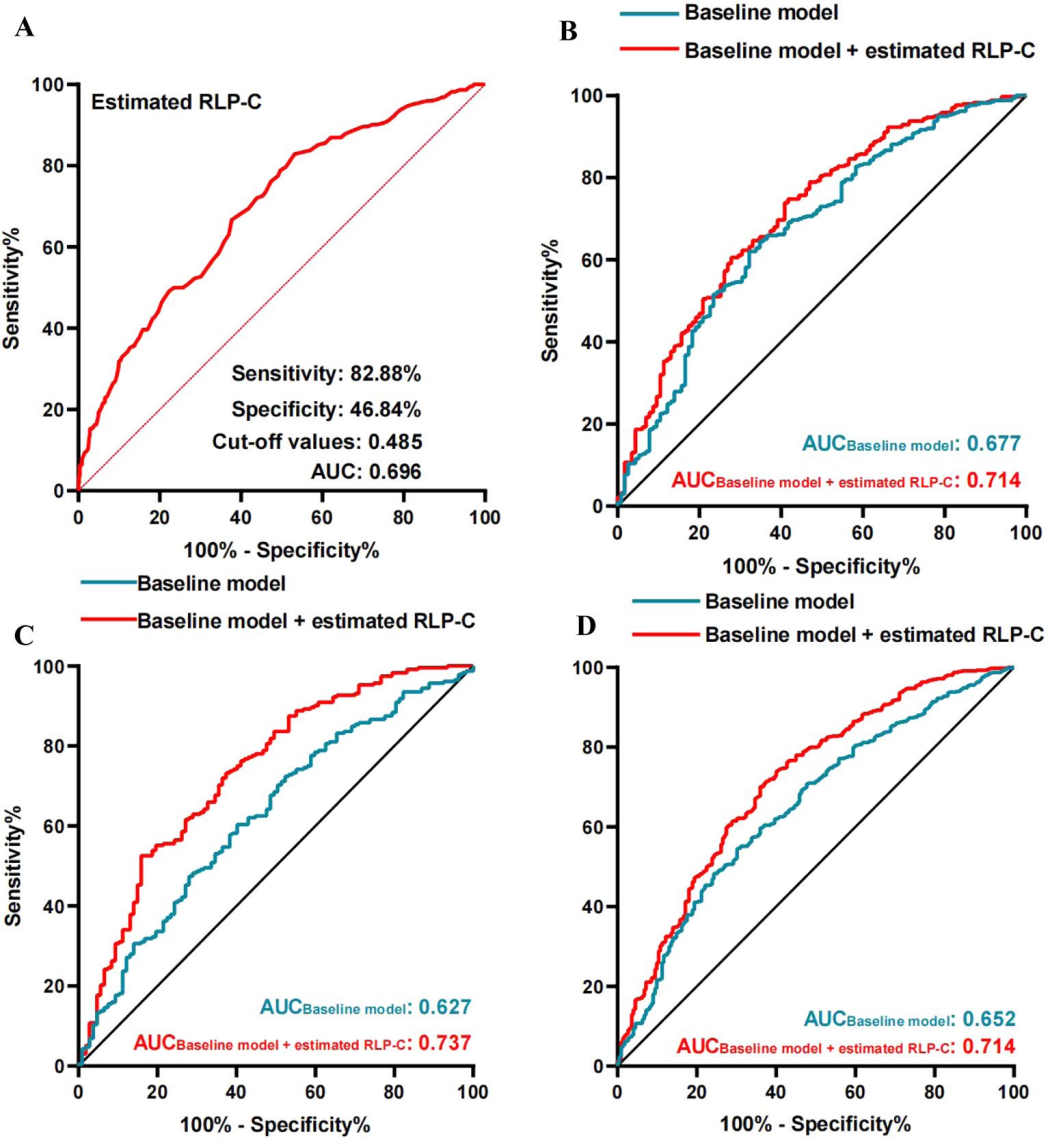
ROC curve of evaluating the diagnostic value of low collateralization in total populations and T2DM subgroups. **A** the diagnostic value of RLP-C for low collateralization in total population; **B** the diagnostic value of baseline model and + the estimated RLP-C for low collateralization in T2DM ( − ) population; **C** the diagnostic value of baseline model and + the estimated RLP-C for low collateralization in T2DM ( +) population; **D** the diagnostic value of baseline model and + the estimated RLP-C for low collateralization in overall population. The baseline model includes: MS, ISR-CTO, LDL-C, HDL-C, HbA1c, Log Lp(a), Log hs-CRP. *ROC* Receiver-operating characteristics, *T2DM* Type 2 diabetes mellitus, *RLP-C* Remnant-like particle cholesterol, *AUC* Area under the ROC curve, *Lp(a)* Lipoprotein(a), *Hs-CRP* High-sensitivity C-reactive protein, *CTO* Chronic total occlusion, *ISR* In-stent restenosis, *LDL-C* Low-density lipoprotein cholesterol, *HDL-C* High-density lipoprotein cholesterol, *HbA1c* Glycated hemoglobin A1c

To further explore the association between the estimated RLP-C and coronary collateralization, multivariate logistic regression analysis was performed. Factors influencing the formation of coronary collateralization were shown in Fig. 2. After adjustment for various confounding factors, the odd ratios (ORs) and 95% confidence intervals (CIs) of the estimated RLP-C for the impaired collateralization were 2.34 (1.46–3.74) and 4.91 (3.01–8.02) in the T2 and T3 group, respectively (Fig. 2A). The correlation between the estimated RLP-C and impaired collateralization was also verified in those with T2DM or not (Fig. 2B and C). The ROC curve in Fig. 3A exhibited the diagnostic value of the estimated RLP-C for assessing the coronary collaterals, with a best cut-off value of 0.485. Figure 3B–D showed an improvement on diagnostic value can be seen when adding the estimated RLP-C into baseline model consisting of MS, ISR-CTO, LDL-C, HDL-C, HbA1c, Log Lp(a) and Log hs-CRP. Further, a significant enhancement in risk reclassification and discrimination was only found in T2DM and overall populations after inclusion of the estimated RLP-C into baseline model, with a category-free NRI of 0.191 (*P* < 0.001), IDI of 0.131 (*P* < 0.001) in the T2DM population and a category-free NRI of 0.100 (*P* < 0.001), IDI 0.070 (*P* < 0.001) in the overall population (Table 2).

**Table 2:** Discriminative value of different models for low collateralization in groups with T2DM or not

	ROC curve analysis				Category-free NRI				IDI		
	AUC	95% CI	P value		index	95% CI	P		index	95% CI	p value
Non-T2DM population											
Baseline model	0.677	0.631–0.720	reference		–	–	reference		–	–	reference
+ estimated RLP-C	0.714	0.670–0.755	0.019		0.064	-0.008–0.136	0.09		0.038	0.017–0.060	< 0.001
T2DM population											
Baseline model	0.627	0.573–0.678	reference		–	–	reference		–	–	reference
+ estimated RLP-C	0.737	0.687–0.783	< 0.001		0.191	0.045–0.204	< 0.001		0.131	0.090–0.171	< 0.001
Overall population											
Baseline model	0.652	0.618–0.685	reference		–	–	reference		–	–	reference
+ estimated RLP-C	0.714	0.681–0.745	< 0.001		0.100	0.044–0.157	< 0.001		0.070	0.048–0.091	< 0.001

*T2DM* Type 2 diabetes mellitus; *RLP-C* Remnant-like particle cholesterol, *ROC* Receiver-operating characteristics, *AUC* Area under the ROC curve, *NRI* Net reclassification improvement, *IDI* Integrated discrimination improvement, *CI* Confidence interval

## Discussion

The major findings of the present study are as follows: (1) The estimated RLP-C level is significantly higher in patients with impaired collateralizations. (2) The increased estimated RLP-C was independently associated with the risk of impaired collateralization in CAD patients with CTO lesions. (3) Adding the estimated RLP-C into the baseline model showed an enhancement on the discrimination of impaired collateralization. (4) The increased estimated RLP-C exhibited a higher risk of forming less-developed collateralization only in total and T2DM populations.

Establishing a well-developed coronary collateralization represents an important therapeutic opportunity for those advanced CAD patients not suitable for vascular revascularization. Studies focusing on validating whether well-developed coronary collateralization could reduce infarcted size, improve cardiac function and decrease long-term MACEs rates have delivered positive results [6, 15, 16]. Adaptative collateral vessel growth often refers to compensatory coronary collateral network development as a result of coronary artery occlusion [23]. Vascular growth in adult organism mainly involves two mechanisms: facilitation of de novo collateral formation (angiogenesis) and the remodeling of pre-existing collaterals through enlarged lumen and thickening vascular walls to provide sufficient blood flow to the regions of heart distal to occluded vessels (arteriogenesis) [6]. The processes of collateral growth are likely to be influenced by microenvironment of coronary circulation (often in the context of T2DM, dyslipidemia or MS) and therefore inducing impaired coronary collateralizations [7, 8]. Factors that are involved in the development of collateral branch include proangiogenic growth factors, endothelial function, inflammatory mediators and the production of reactive oxygen species (ROS) [24]. Using angiogenic growth factors as therapeutic stimulation of coronary collateral network in animal models has achieved promising results and significant therapeutic benefits with increased regional cardiac perfusion in the past two decades [25, 26]. However, the progress of proangiogenic therapy achieved in preclinical studies failed to deliver positive results in clinical practice and cannot improve CAD patients’ cardiac function and long-term prognosis [27]. Reasons underlying this discordance between the results angiogenic growth factor have achieved in animal models and CAD patients can be partly due to the different characteristics of CAD patients and animal models. CAD patients in clinical settings are generally older and more likely to have more cardiovascular risk factors and clinical morbidities like diabetes, MS and chronic renal dysfunction [28–30], which may result in impaired CCG and reduced responsiveness to biochemical factor therapy. The present study demonstrated that increased estimated RLP-C is independently associated with less developed collaterals, indicating the value of estimated RLP-C in discriminating the formation of collateralization. Besides, estimated RLP-C could provide a new insight into understanding the mechanism of coronary collateral growth in clinical settings. Evidence from former studies found that elevated remnant lipoprotein cholesterol rather than cholesterol in the LDL-C is causally associated with systemic low-grade inflammation marked by the increase of CRP and that association is independent of low HDL-C [13]. One likely explanation could be that cholesterol content of remnant lipoprotein carry more cholesterol and can be directly taken up by macrophages while LDL-C has to be oxidated as oxLDL-C to elicit inflammation [31]. Moens et al. found that 1.2fold increase in 18F-FDG uptake in the arterial wall for every 1 mmol/L increase in remnant cholesterol, indicating a correlation between remnant cholesterol and arterial wall inflammation [32]. Estimated RLP-C reflects angiographic coronary collateralization could also be explained by endothelial dysfunction. Nakamura et al. found an improvement of vascular flow-mediated dilation after the treatment with statin therapy in CAD patients and this improvement is associated with the reduction of RLP-C. RLP-C was also validated as an indicator of endothelial vasomotor dysfunction [33]. In Vitro study found that RLP-C could accelerate endothelial progenitor cells senescence via increased production of ROS and decrease adhesion, migration and proliferation capacities of endothelial progenitor cells [34]. Hence, the association between the estimated RLP-C and impaired collateralizations could be partly explained in terms of inflammation and endothelial dysfunction.

Former studies reported impaired coronary collateral growth in abnormal metabolic status like T2DM and MS [7, 8], while the value of HbA1c and MS in discriminating impaired collateralization seems weakened after adjustment in current study (Fig. 2). This can be partly attributable to the robust associations between the estimated RLP-C and metabolic abnormality. Previous studies have revealed that the RLP-C level is more prominent in patients with abnormal metabolic status [14]. T2DM is characterized by impaired lipoprotein metabolism, comprising elevations in TRL and decreased levels of HDL-C [35]. Although most patients in current study were all taking standard statin therapies (83%), the estimated RLP-C level in T2DM population is still significantly higher than its counterpart without T2DM. Further, an enhancement in discriminative value of impaired collateralization can be only seen when incorporating estimated RLP-C into baseline model in patients with T2DM (Fig. 3 and Table 2), which indicated that there exist interactive effects between the estimated RLP-C and glycometabolic status on the development of collateral branch. Hence, therapies targeting at RLP-C may be warranted in those T2DM patients with extremely high cardiovascular risks. Hatori [36] has demonstrated that empagliflozin could significantly decrease the inflammation level in T2DM patients and this alteration could be partly attributed to the decrease of remnant lipoprotein, indicating the potential of sodium-dependent glucose transporters 2 (SGLT2) inhibitors in ameliorating the coronary collateral growth and improving the prognosis of those patients. Further trials evaluating the impact of SGLT2 inhibitors in coronary collateralization are necessary.

Another notable finding in current study showed that Lp (a) was also significantly associated with the under-developed coronary collateralization after adjusting for various factors (OR 1.41, 95%CI 1.03–1.91, *P* = 0.03), which is in accordance with previous findings [37, 38]. The mechanism underlying could be partly attributable to the Lp(a) induced endothelial dysfunction and inflammation[39, 40], which is similar to the mechanism of RLP-C as discussed above. Morishita et al. [39] found that high serum Lp (a) impaired the collateral growth of the Lp(a) transgenic mice in the event of hindlimb ischemia via inhibiting the transforming growth factor-β activity, suggesting the detrimental role of Lp(a) in collateral vessel development. However, the current study failed to find a positive relationship between Lp (a) and low collateralization in those T2DM patients (OR 1.23 95%CI 0.78–1.96, P = 0.376), which is contradicted with the previous study by Shen et al.[37]. The following reasons could account for this disparity: (1) the enrolled populations in previous study were stable CAD patients with CTO lesions, suggesting the types of CAD may be a determinant of collateral development in clinical practice. (2) Considering the similar mechanism of Lp (a) and RLP-C in interfering with collateral growth, the weakened discriminative role of Lp (a) in T2DM patients could be partly explained by the increased association between estimated RLP-C and collateral development [OR (95%CI) 4.95(2.35–10.45) and 10.58 (4.77–23.45) in the T2 and T3 group, *P* < 0.001], indicating RLP-C could exert greater effect on the formation of coronary collateralization than Lp (a), especially in those T2DM patients.

Several limitations should be acknowledged: (1) The design of this study may limit the generalizability of the results. (2) Although the established risk factors influencing the development of collateralization were statistically adjusted using multivariate logistic regression models, this adjustment cannot entirely remove the effect of other confounding factors, like proangiogenic factors, inflammatory cytokine, etc. (3) More detailed medical history influencing the estimated RLP-C level like fibrates and glucose-lowering therapies of T2DM patients is scarce in this study. (4) The lack of data about WC could impair the accuracy of diagnosing MS, thus weakening the efficacy of the conclusions when incorporated MS into the multivariate logistic regression model. (5) The estimation of collateral grading could be more accurately assessed by coronary flow index [41] but the Rentrop scoring system is easier to use in clinical practice. (6) Although the formula used to calculate the RLP-C can be affected by chylomicrons especially in the non-fasting state and the last meal’s fat content in current study, it is easy to use and readily accept in large population studies with no additional expense [42].

In conclusion, the estimated RLP-C is independently associated with impaired collateralization in T2DM patients with coronary CTO lesions. Mechanism underlying this association need to be further investigated and therapies targeting at the estimated RLP-C may be needed in advanced CAD patients with T2DM not suitable for vascular revascularization.

## Data Availability

The dataset and materials mentioned above are available from the authors.

## Abbreviations

CTO: Chronic total occlusion
CAD: Coronary artery disease
PCI: Percutaneous coronary intervention
MACEs: Major adverse cardiovascular events
CKD: Chronic kidney disease
CCG: Coronary collateral growth
T2DM: Type 2 diabetes mellitus
MS: Metabolic syndrome;
RLP-C: Remnant-like particle cholesterol
TG: Triglyceride
TRL: Triglyceride-rich lipoprotein
ASCVD: Atherosclerotic cardiovascular disease
LDL-C: Low-density lipoprotein cholesterol
MI: Myocardial infarction
NYHA: New York Heart Association
LVEF: Left ventricular ejection fraction
HTN: Hypertension
FBG: Fasting blood glucose
HbA1c: Glycated hemoglobin A1c
TCHO: Total cholesterol
HDL-C: High-density lipoprotein cholesterol
LDL-C: Low-density lipoprotein cholesterol
WC: Waist circumference
BMI: Body mass index
GA: Glycated albumin
Hs-CRP: High-sensitivity C-reactive protein
ROC: Receiver-operating characteristics
AUC: Area under the ROC curve
IDI: Integrated discrimination improvement
NRI: Net reclassification improvement
RCA: Right coronary artery
LAD: Left anterior descending artery
OR: Odds ratio
CI: Confidence interval
ROS: Reactive oxygen species
SGLT2: Sodium-dependent glucose transporter 2
